# Novos Marcadores de Espessamento Carotídeo na Hipertensão Arterial

**DOI:** 10.36660/abc.20201335

**Published:** 2021-01-27

**Authors:** Rui Póvoa

**Affiliations:** 1 Universidade Federal de São Paulo São PauloSP Brasil Universidade Federal de São Paulo, São Paulo, SP – Brasil

**Keywords:** Hipertensão/epidemiologia, Monócitos, mRNA, Espessura Intima Media Carotidea, Marcadores Genéticos

A hipertensão arterial foi considerada como importante fator de risco cardiovascular somente após os estudos de Framingham, pois se acreditava que era um “bem” necessário para uma boa perfusão tecidual.^1^ Estes estudos emblemáticos de coorte em longo prazo sobre o sistema cardiovascular trouxeram dados que permitiram avaliar a interação de diversas outras doenças, tais como as dislipidemias e o diabetes, para a formação da placa de ateroma que é o passo inicial para as complicações cardiovasculares. Época em que o exame clínico era fundamental para detectarmos os marcadores de doença aterosclerótica.

Porém, com a evolução do conhecimento, os marcadores biológicos clínicos já não eram suficientes para prever o risco, pois precisamos cada vez mais articular medidas preventivas o mais precoce possível para um tratamento mais eficaz e uma melhor prevenção. Além disso, a interação meio ambiente com todos os seus fatores de risco e a genética se mostraram interativos e de fundamental importância no desenvolvimento da placa aterosclerótica. Para o lado da hipertensão ficou claro o componente genético com estimativa de herança de 15-40%, tanto que os irmãos apresentam uma taxa de concordância de risco para a doença com variações de 1,2 a 1,7. ^2 , 3^

Para entendermos este mecanismo extremamente complexo, que envolve diversas vias moleculares e bioquímicas, tais como o sistema renina angiotensina aldosterona (SRAA), intimamente ligado à hipertensão, a análise de marcadores biomoleculares e/ou genéticos pode agregar conhecimento para desvendar as diversas vias que levam a aterosclerose.

Os fatores étnicos e raciais também contribuem, predispondo a maior prevalência de diversas doenças e entre elas a hipertensão. Exemplo disso são os afrodescendentes e os latinos com maior prevalência e gravidade, além de acentuação das comorbidades relacionadas à doença hipertensiva.^4 , 5^

O objetivo do trabalho de Gamboa et al.,^6^ foi avaliar a associação de marcadores biomoleculares e genéticos com a hipertensão arterial focando principalmente a espessura intima-média de carótida (EIMC) em mexicanos.^6^

A população mexicana tem uma mistura étnica de 65% de índios americanos, 31% de europeus, e 3% de africanos diferindo muito de outros países com predominância caucasiana, onde a maioria dos estudos é feita.^7^Esta carga genética diversificada pode levar a um comportamento específico em termos de risco cardiovascular e de expressão de marcadores.

O EIMC que é um marcador de aterosclerose, correlaciona-se com um aumento de mortes e eventos cardiovasculares em adultos e também com anormalidades vasculares em crianças e adolescentes hipertensos.^8^Lande et al. observaram que crianças ou adolescentes com EIMC acima do normal apresentavam hipertensão mais grave, e era independente da obesidade usualmente relacionada, nesta faixa etária, com a doença hipertensiva.^9 , 10^

Gamboa et al.,^6^ encontraram no grupo de hipertensos valores maiores do EIMC e relacionaram ao aumento na expressão mRNA de LRP1 e a expressão de proteína LRP1 a qual apresentou valores elevados e muito evidentes nos hipertensos.

Os mecanismos pelo qual a hipertensão predispõe a aterosclerose ainda não estão bem esclarecidos, porém sabe-se que é multifatorial envolvendo diversas causas, desde aspectos endoteliais, lipídicos e genéticos. Entretanto a EIMC também aumenta como reação fisiológica vascular em adaptação ao aumento pressórico e ao progredir dos anos refletindo resposta adaptativa ao envelhecimento e ao estresse mecânico. Estes achados são interessantes mostrando estes marcadores mais elevados naqueles hipertensos com maior EIMC. Isto corrobora a teoria multifatorial da hipertensão e das lesões em órgãos-alvo, onde o perfil genético influencia profundamente a agressão vascular.^11 , 12^Fato também encontrado em estudos experimentais em animais que mostraram que a LRP1 promove a entrada de lípides em monócitos que migaram ao vaso formando as células espumosas e assim a aterosclerose.^13^

Achado curioso foi na divisão dos grupos pelo sexo. A expressão mRNA de LRP1 em hipertensos foi significantemente maior em mulheres e pouco expressivo no homem, que foi praticamente igual aos normotensos. Isto dificulta o entendimento, pois carece de uma explicação objetiva desta diferença. Fato que não ocorreu na expressão de proteína LRP1, que se elevou no grupo hipertenso de forma similar em homens e mulheres. A média de idade dos hipertensos foi de 50,3 anos e possivelmente fatores hormonais ligados ao sexo feminino talvez possam estar envolvidos.

Neste estudo foi avaliado a angiotensina II (Ang II), visto a importância do SRAA na regulação da hipertensão. Verificaram uma relação positiva da Ang II com a expressão de LRP1, relacionando a pressão elevada como regulador da expressão de LRP1 mediado pela Ang II.

O SRAA é muito complexo e cada vez mais amplia os conhecimentos, pois novos dados são acrescentados na didática cascata bioquímica que se inicia com a renina e finaliza na aldosterona. A complexidade é tanta que o bloqueio único com os inibidores da enzima conversora da angiotensina ou dos bloqueadores AT1 da Ang II promovem benefícios clínicos fantásticos, entretanto o bloqueio duplo apresenta resultados pífios ou até maléficos ao paciente. De tal forma, conclusões óbvias baseadas em aspectos fisiopatológicos não se aplicam plenamente para o SRAA.

O estudo apesar de complexo em análise dos dados, é um caminho para o desenvolvimento de novos marcadores em hipertensão arterial que possam nos guiar na procura de lesões a órgãos-alvos precoces que será traduzido em uma terapêutica mais precisa com metas mais otimizadas, beneficiando o paciente.
