# Supplementary material for: Novos Marcadores de Espessamento Carotídeo na Hipertensão Arterial
Source: Arq Bras Cardiol. 2021 Jan 27;116(1):66–7. [Article in Portuguese] doi: 10.36660/abc.20201335 (PMC8159507; doi:10.36660/abc.20201335)
Supplement: Translation

# Translation: New Markers of Carotid Thickening in Hypertension

**Authors:** Rui Póvoa

**Affiliations:** 1 Brazil Universidade Federal de São Paulo, São Paulo, SP – Brazil

**Keywords:** Hypertension/epidemiology, Monocytes, mRNA, Carotid Intima-Media Thickness, Genetic Markers

Arterial hypertension was considered an important cardiovascular risk factor only after the Framingham studies, as it was believed that it was a necessary “good” for good tissue perfusion.^1^These emblematic long-term cohort studies about the cardiovascular system brought data that allowed the evaluation of the interaction with several other diseases, such as dyslipidemias and diabetes, for the atheromatous plaque formation, which is the initial step for cardiovascular complications. A time when clinical examination was essential to detect markers of atherosclerotic disease.

However, with the evolution of knowledge, clinical biological markers were no longer sufficient to predict risk, as we increasingly need to articulate preventive measures as early as possible, for more effective treatment and better prevention. Additionally, the interaction between the environment, with all its risk factors and genetics proved to be interactive and of crucial importance in the development of the atherosclerotic plaque. Regarding hypertension, the genetic component with an estimated inheritance of 15-40% became clear, so much so that the brothers have a risk agreement rate for the disease ranging from 1.2 to 1.7.^2 , 3^

To understand this extremely complex mechanism, which involves several molecular and biochemical pathways, such as the renin-angiotensin-aldosterone system (RAAS), closely linked to hypertension, the analysis of biomolecular and/or genetic markers can add knowledge to reveal the several pathways that lead to atherosclerosis.

Ethnic and racial factors also contribute to it, predisposing to a higher prevalence of several diseases, including hypertension. An example of this fact are Afro-descendant and Latin populations, with greater disease prevalence and severity, in addition to more marked comorbidities related to hypertensive disease.^4 , 5^

The objective of the study by Gamboa et al.^6^was to evaluate the association of biomolecular and genetic markers with arterial hypertension, focusing mainly on the carotid intima-media thickness (CIMT) in Mexicans.^6^

The Mexican population has an ethnic mix of 65% American Indians, 31% Europeans, and 3% Africans, differing greatly from other countries with a predominance of Caucasians, where most studies are performed.^7^This diverse genetic load can lead to a specific behavior in terms of cardiovascular risk and marker expression.

The CIMT, which is a marker of atherosclerosis, correlates with an increase in deaths and cardiovascular events in adults and also with vascular abnormalities in hypertensive children and adolescents.^8^Lande et al. observed that children or adolescents with CIMT that was above normal values had more severe hypertension, irrespective of obesity, usually associated to hypertensive disease in this age group.^9 , 10^

Gamboa et al.^6^found higher CIMT values in the hypertensive group and associated it to an increase in LRP1 mRNA expression and the expression of LRP1 protein, which showed high and very evident values in hypertensive patients.

The mechanisms through which hypertension predisposes to atherosclerosis are not yet well understood, but it is known that they are multifactorial involving several causes, from endothelial aspects, to lipid and genetic ones. However, CIMT also increases as a physiological vascular reaction in adaptation to pressure increase and as the years progress, reflecting an adaptive response to aging and mechanical stress. These findings are interesting, demonstrating that these markers are higher in hypertensive patients with higher CIMT. This corroborates the multifactorial theory of hypertension and target-organ injury, where the genetic profile profoundly influences vascular injury.^11 , 12^A fact also found in experimental studies in animals that showed that LRP1 promotes the entry of lipids in monocytes that migrated to the vessel forming foam cells and, therefore, atherosclerosis.^13^

A curious finding was related to the division of groups by gender. The mRNA expression of LRP1 in hypertensive individuals was significantly higher in women and less significant in men, which was practically the same as in normotensive individuals. This makes understanding difficult, as it lacks an objective explanation of this difference. This did not occur in the expression of LRP1 protein, which increased in the hypertensive group in a similar manner in men and women. The mean age of hypertensive patients was 50.3 years and, possibly, hormonal factors related to the female gender may be involved.

In this study, angiotensin II (Ang II) was evaluated, considering the importance of RAAS in hypertension regulation. They found a positive relationship between Ang II and LRP1 expression, associating high pressure as a regulator of LRP1 expression mediated by Ang II.

The RAAS is very complex and knowledge about it has been increasingly expanding, as new data is added to the didactic biochemical cascade that begins with renin and ends with aldosterone. The complexity is such that the single block with angiotensin-converting enzyme inhibitors or Ang II AT1 blockers promotes fantastic clinical benefits; however, the double block has poor or even harmful results for the patient. Thus, obvious conclusions based on pathophysiological aspects are not fully applicable to RAAS.

The study, although complex regarding the data analysis, is a way for the development of new markers in arterial hypertension that can guide us in the search for early target-organ injuries that will be translated into more accurate therapy, with more optimized goals, benefiting the patient.
